# Should We Use Clinical Tools to Identify Disease Progression?

**DOI:** 10.3389/fneur.2020.628542

**Published:** 2021-01-21

**Authors:** Hernan Inojosa, Undine Proschmann, Katja Akgün, Tjalf Ziemssen

**Affiliations:** Multiple Sclerosis Center, Center of Clinical Neuroscience, Department of Neurology, University Hospital Carl Gustav Carus, Dresden University of Technology, Dresden, Germany

**Keywords:** multiple sclerosis, disease progression, secondary progressive multiple sclerosis (SPMS), clinical diagnosis, pathophisiology

## Abstract

The presence of disability progression in multiple sclerosis (MS) is an important hallmark for MS patients in the course of their disease. The transition from relapsing remitting (RRMS) to secondary progressive forms of the disease (SPMS) represents a significant change in their quality of life and perception of the disease. It could also be a therapeutic key for opportunities, where approaches different from those in the initial phases of the disease can be adopted. The characterization of structural biomarkers (e.g., magnetic resonance imaging or neurofilament light chain) has been proposed to differentiate between both phenotypes. However, there is no definite threshold between them. Whether the risk of clinical progression can be predicted by structural markers at early disease phases is still a focus of clinical research. However, several theories and pathological evidence suggest that both disease phenotypes are part of a continuum with common pathophysiological mechanisms. In this case, the clinical evaluation of the patients would play a preponderant role above destruction biomarkers for the early identification of disability progression and SPMS. For this purpose, the use of clinical tools beyond the Expanded Disability Status Scale (EDSS) should be considered. Besides established functional tests such as the Multiple Sclerosis Functional Composite (MSFC), patient's neurological history or digital resources may help neurologists in the decision-taking. In this article, we discuss arguments for the use of clinical markers in the detection of secondary progressive MS and the characterization of progressive disease activity.

## Introduction

Multiple sclerosis (MS) is the most common chronic autoinflammatory disease of the central nervous system (1). In its disease course, most of the patients present an initial relapsing remitting course (RRMS), where clinical relapses or attacks with a complete or partial recovery of neurological symptoms are the hallmark. Many RRMS patients develop secondary progressive disease (SPMS) with disability progression independent of relapses. The identification of this progression in MS is evolving aside from the new recently available therapeutic options, although significant challenges and problems still represent diagnostic difficulties (2). So, the transition phase from the relapsing to the secondary course is still difficult to define or predict.

In MS, the term progression is used in the context of neurological functional disability. This is defined in the latest revisions of the McDonald criteria for MS, which include symptomatic fluctuations as part of this complex disease course (3). In the last years, the knowledge about this disease phase has been deepened, supporting the analysis of data in recent clinical trials (4). The concept of “silent progression” has been recently proposed, whereas the recognition of the neurodegenerative process underlying the insidious disability progression in relapsing MS patients is difficult to achieve (e.g., in patients with lower disability scores) (5). An additional distinction has been conceptualized in RRMS patients regarding the influence of relapses on their functional disability, emerging from the definitions of progression independent of relapse activity (PIRA) and relapse-associated worsening (RAW), which could support the identification of disease progression in clinical trials (6, 7). Moreover the confirmation of disability over time (e.g., in a period of 3 or 6 months, or more) is considered in the definition of confirmed disability progression (CDP), which may englobe the PIRA and RAW as well, when the influence of relapses in their recovery is considered (7, 8).

Several compensatory mechanisms can mask these gradual changes, making the diagnosis of progression difficult (2, 5, 9). The degree at which the MS complaints affect neurological function may also vary according to several non-MS-related external and internal factors including, e.g., the weather, emotions, or stress. Thus, the objective identification of clinical disability and its effect on daily functioning are in sharp focus in the current definitions of SPMS and should also be the focus in clinical practice.

However, in the setting of MS, even though certain biomarkers suggest differences in the profiles of RRMS, PPMS, and SPMS patients, there is still not enough pathological evidence of clear differences between each disease phenotype (10). The only difference related to structural markers seems so far to be more at a quantitative than qualitative level (11). The neuro- and immunopathological structural changes are apparently common in all MS subtypes with individual differences of their contribution to the clinical phenotype rather due to the burden of these lesions than to different pathological characteristics (11).

The actual gold standard used for demonstrating clinical progression is the neurological examination as documented by the Expanded Disability Status Score (EDSS) (12). The EDSS has been widely criticized due to several psychometric limitations including a low responsiveness especially at upper levels of the scale (4). Other clinical tools such as the Multiple Sclerosis Functional Composite (MSFC) and its subtests are almost exclusive for specialized MS centers or the setting of clinical trials. Other clinical measures to identify clinical progression in the management of people with MS are therefore lacking.

In this short review, we discuss the use of clinical parameter for the identification of progression based on pathophysiological considerations in order to support the early recognition of transitioning patients from RRMS to SPMS.

## Pathophysiology of Disease Progression

While actively demyelinating plaques are classically more associated with patients with RRMS, brain atrophy is considered a pathological finding more typical of the secondary progressive disease course (13, 14). However, e.g., brain atrophy is already present in early disease stages of RRMS patients (15). The use of disease-modifying therapies (DMTs) seems to attenuate the further development of brain atrophy and consequently the development of disability progression (16–19). In untreated or placebo-treated patients, brain atrophy developed at an increased rate compared to those on DMTs, suggesting that this process begins already on this phase (17). So, the development of brain atrophy is not a characteristic element of the SPMS phenotype as well.

Processes of gradual neuro- and immunosenescence seem to be already present in RRMS patients. Several immunological cascades including activation of peripheral immune cells, cytokine/protease secretion, increase of the blood–brain barrier permeability, and perivascular infiltration of lymphocytes may be responsible for neuroglial injury and neuronal degeneration (20). Immunological alterations seen in SPMS may be driven by the chronic inflammation already present in RRMS. The clinical transition from RRMS to SPMS occurs with an underlying change in the immunopathological components of the disease. One hypothesis suggests that the exhaustion of resources through aging and lesion accumulation is responsible for clinical progression (20). Alternatively, proper chronic demyelination of SPMS may occur after reaching a variable inflammatory threshold, where the environment that supports this chronic inflammation produces a neuronal dysfunction with neurotransmitter disbalance in a vicious cycle (20).

**Figure 1:**
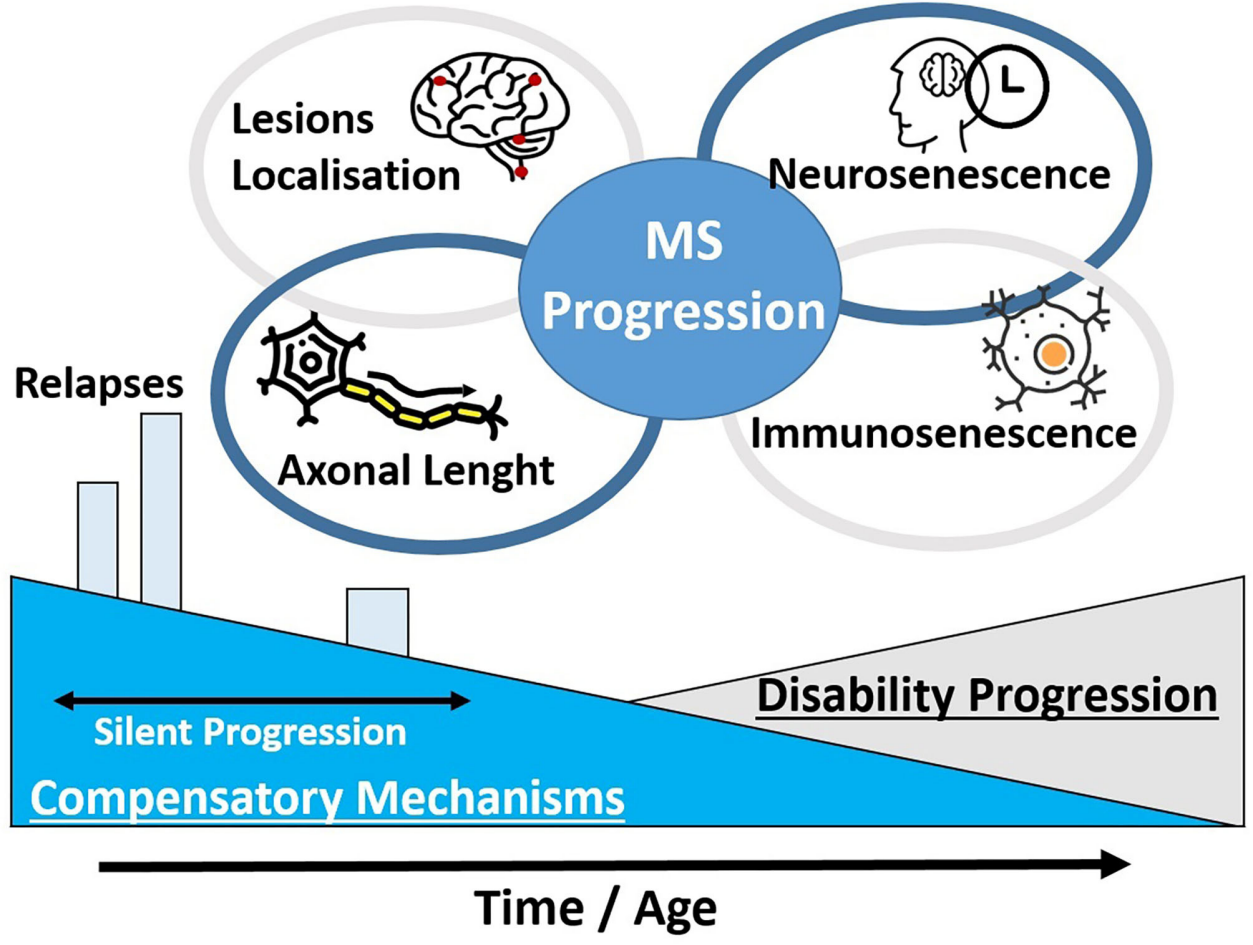
Certain pathophysiological theories for the clinical disability progression in MS patients are unified. The development of clinical disability may depend at a great part on the decrease of compensatory mechanisms and an advance in the patients' age. In addition, the localization of MS lesions and the axonal length of these domains may explain the different clinical presentation among MS patients. A progressive neurosenescence and immunosenescence process may occur in a phase of silent progression.

In addition to directly disease-specific progression, older MS patients may present with age-related alterations such as synaptic dysfunction and plasticity impairment (21). These alterations could account for silent progression until individual thresholds were reached and these processes became clinical apparent (Figure 1) (20). An overlap between expected neurological function impairment in older patients and the disease-related process is expected to occur, representing difficulties in the classification of symptoms and complaints.

In line with these observations from pathology and immunology, RRMS and SPMS seem to be part of a continuum of a common pathophysiological process. According to different functional compensatory mechanisms, patients may express the classical forms of disease course at a different rate of clinical disease progression (22, 23). Progressively, active inflammatory lesions may account for a degenerative axonopathy, which would clinically lead to disability progression (24). Damage assessed by NfL is observed already in early stages of RRMS patients who develop secondary disability progression in the disease course (25). Likewise, it has been suggested in a French cohort that the onset of disability is at a great part an age-related process as the onset age of the PPMS and the diagnosis of SPMS are done at a very similar age (22, 26). In this line, it has been demonstrated that at a more advanced age, relapses may have a lower impact on the accumulation of disability and the onset of SPMS could be at last a time-related process (21, 27–29). The remyelination of MS lesions would decrease over time as well (30). However, the current use of DMTs in RRMS patients may delay the onset of secondary progression altering the untreated disease course as recent studies suggest (18).

The topographical model of MS by Krieger and colleagues unifies both disease courses and the transition phase to describe the clinical course of MS; clinical disability progression manifests as reduction in functional reserve resulting in the unmasking of silent pathological findings (23). This model considers the dynamic factors that underlie the individual MS phenotype including its clinical manifestation. The regional localization of lesions and the functional capacity of the individual patient, which can be affected by inherent or by external factors, determines the progression in defined functional neurological domains as, e.g., motor or visual function. A key element in this model is the hypothesis that MS progression reveals rather older demyelinating processes present already in early disease phases. Among MS heterogeneity, this model presents five relevant factors: localization of lesions, frequency, severity and recovery of relapses, and brain volume at baseline and progression.

Another interesting theory, which was suggested at first by Kurtzke and deepened by Giovannoni et al., questions as well the MS phenotypes based on the axonal length (8). According to this model, the inflammatory demyelination would manifest earlier and to a greater extent in axonal pathways with longer axons compared to pathways with shorter projections, which are more resilient (e.g., lower limbs vs. facial nerve). Dysfunction involving domains with longer axonal projections could predict poorer outcomes and progressive disease as these could accumulate more with increasing demyelinating lesions. Shorter axons (e.g., upper limb, brain stem, or visual) are frequently less involved at initial phases of progressive MS (31, 32). This model also suggests that the smaller size of motor units with larger corresponding areas of the cortex may serve as a protective factor for progression of hand or arm motor function for example (32). The application of tests to evaluate functions in these domains beyond ambulation could be considered not only to detect progression but also to monitor treatment effect in domains with shorter axonal projections.

These studies support a rethinking about MS classifications and phenotypes. All phenotypes may be part of the same disease continuum starting with a radiological or clinically isolated syndrome. Factors such as the localization (and burden) of lesions, the brain reserve, and other compensatory mechanisms (which are at a great part age-related) would be the key in the clinical manifestation of disease (5).

In consonance with these ideas, a recent clinical study showed that even in a cohort composed exclusively of RRMS patients, most of the disability accumulation was independent of relapses (8). This could even be observed using the EDSS—which is known for several limitations—supported by changes in two functional tests, the timed 25-feet walk (T25FW) and the nine-hole peg test (9HPT). Using clinical outcome measures with a greater responsiveness would almost certainly support and reinforce this analysis (9).

Naturally, certain risk factors have been described, which could suggest or predict the manifestation of secondary progression in MS patients. Among the demographic characteristics of the patients at the RRMS diagnosis, male sex (29, 33–35), older age (21, 29, 35–38), and a multifocal disease manifestation at onset (36, 37) have been associated with an earlier transition to SPMS. Regarding the disease course, patients with a low EDSS at MS diagnosis could have a lower risk of disability progression further on (36); the annual relapse rate may be a predictive factor especially in the earlier disease stages (27, 29, 35, 36). Similarly, the presence of gadolinium enhancement and spinal cord lesions in the first 3 years of the disease have been associated with the development of SPMS within 15 years (39). The identification of NfL could play an interesting role in detecting disease activity and further neurological damage as already stated above (40). A recent study correlated high sNfL at early stages of the disease with an increased risk of disability progression in the next 15 years (25).

The use of DMTs seems to modify the risk of a secondary progressive course. First-line DMTs may prolong the period to transition to SPMS according to several studies (18, 19, 29, 36), although a lack of an objective influence has also been suggested (37, 41). Furthermore, the use of newer high-effective DMTs is associated with an even lower risk of disability accumulation compared to the first-line therapies (19). Conversely, a recent study with a large cohort of MS patients could not confirm an association of brain or spinal cord MRI evidence of disease activity with the risk of SPMS (29). However, in line with the age of diagnosis, a longest disease duration may be one important and unfortunately non-modifiable risk factor for secondary progression (29, 35).

So, even though the radiological disease burden may support and suggest the probability of developing clinical disease progression, it seems currently impossible to identify the transition phase to SPMS through imaging or neurodestruction biomarkers. A more intensive disease activity (clinical, radiological, or eventually assessed by sNfL) in early RRMS may suggest a higher chance of early SPMS development; however, a prediction is not possible to date. For this reason, the classical assessment of the neurological status through clinical outcomes may be in the spotlight when it comes to identifying disability progression. Imaging resources are significant tools for an objective assessment of the disease course and in the detection of subclinical activity, with the crucial identification of newer or size-progressive lesions. However, as commented above, it should always be interpreted together with clinical outcomes.

## Structural Markers to Identify MS Progression

The use of structural markers is currently intensively investigated for the identification of progression in SPMS and its transition phase (11). Newer MRI techniques have evolved in the last years as well. The precision and quality of the measurements have experienced an exponential increase through the advances in hardware technologies. The quantification of brain atrophy has a good correlate with clinical measures and is one of the most important imaging markers used in progressive MS (5, 42). The volume of T2 hyperintense or T1 hypointense lesions and newer or enlarging lesions are also widely used MRI markers that could even predict the transition to RRMS in certain patients, especially in clinically isolated syndrome (CIS) (11, 43). The development of infratentorial and spinal cord lesions in the disease course is correlated with an increased risk of developing disability progression (39, 44). Beyond these widely used markers, the assessments of the microglial activity, chronic permeability of the blood–brain barrier, or leptomeningeal inflammation promise a novel approach (45). Iron has been demonstrated at the rim of active macrophages and microglia in active MS lesions and could be detected with specific-weighted MRI. The use of positron emission tomography (PET) scans can serve as an alternative method to an *in vivo* evaluation of cellular activation. Alternatively, fluid-attenuated inversion recovery (FLAIR)-MRI is under evaluation for the assessment of meningeal inflammation (45). Automatized software applications, including artificial intelligence, are currently being investigated and may represent a next step in the follow-up monitoring of MS patients.

Other markers are also currently under research. Optical coherence tomography (OCT) with the assessment of the retinal fiber layer (RNFL) and the macular ganglion cell layer may also be a tool to characterize SPMS. This tool is still not established in clinical practice; out of specialized centers, however, there is evidence that even without history of optic neuritis, a progressive RNFL thinning occurs in progressing patients (46, 47). Additionally, SPMS patients seem to have a lower RNFL thickness than RRMS patients (48, 49). A correlation with brain atrophy has also been described for this marker (50). Nevertheless, history of optic neuritis plays, however, an important role and further longitudinal analysis with large cohorts is needed as, on the other hand, certain studies could not demonstrate significant utility for SPMS patients (51). The presence of pathological alterations already on RRMS patients could support, though, the theory of a disease continuum from the beginning of the disease. The extension of a predictive or diagnostic value must be further confirmed. The OCT may present an interesting non-invasive resource to support the detection of clinical progression, although its clinical utility must be further assessed (52).

Beyond imaging, other structural biomarkers are under development and validation in progressive MS. Neurofilament light chain (Nfl) is a novel marker that can be detected in serum or in cerebrospinal fluid with a good correlation between both measures (40, 53, 54). This non-specific biomarker serves as evidence of neuronal destruction and has a good correlation with MRI measures and possible with the risk of developing SPMS (25, 55, 56). However, technical methods for the analysis and Nfl concentrations in healthy subjects and at early phases of the disease should be addressed (56). Future research should focus on the use of this biomarker for the monitoring of treatment response and disease progression.

The glial fibrillary acid protein (GFAP), which suggests astrocyte activity, has been correlated to Nfl levels and could reflect differences between progressive and relapsing MS patients (57). Other protein biomarkers, such as myelin oligodendrocyte glycoprotein or the neurodegenerative tau protein, differed between control and MS patients as well as between MS subtypes (58).

In the setting of acute clinical disease activity, the use of MRI serves as a support in the differential diagnosis of pseudo-relapses or clinical attacks, especially in cases of diagnostic uncertainty with stress factors (e.g., weather, infections) (59). Nfl could possibly offer a similar applicability in a future after further validation of this marker (60–62). Intervals of clinical stability can also be monitored applying these markers to detect subclinical activity and evaluate the indication of therapeutic switch considering a more preventive approach to avoid further inflammatory activity (63).

However, at the time of diagnosing MS progression, the use of these markers may be carefully considered. Although they represent an important support in the diagnosis and monitoring of MS patients, these are not specific for any disease subtype. They indicate as far as evidence reports rather a threshold and probability than a confirmed disability progression. They may indicate acute phases of disease activity, but not a chronically progressive disability in MS patients.

A possible reason seems to be the common pathophysiological process from CIS over RRMS to SPMS. Possibly, neurodegeneration markers (e.g., GFAP) could be a promising complement in the future evaluation of SPMS patients, as astrogliosis could play a different role in advanced stages of the disease.

## Clinical Assessment of Disability Progression

In line with the wide heterogeneity of the MS, the progression of disability is a multidimensional process difficult to detect. Patients and physicians experience several challenges in the objective diagnosis of SPMS (2). Function limitation in MS can affect every neurological domain from gait to bowel function. As commented above, gait and balance dysfunction play a predominant role for MS patients. These functions can condition the daily activities of people with MS. Furthermore, an impairment in these domains can derive in falls (occasionally with trauma), social limitations, or physical complications, such as muscle atrophy and spasm.

As the EDSS final step score, which is key for the only standardized definitions of secondary progression (64), depends on a great magnitude of the ambulation, it is predictable that a gait impairment is present on patients with confirmed disability progression and higher EDSS scores. Up to 93% of a group of SPMS patients reported balance or walking difficulties as a symptom (33). The evaluation of balance could be supported by specific posturography tests, which could detect disability already on early disease phases and with a higher reliability than rather-dependent subjective evaluations (65).

Fatigue and spasm complete the most common complaints in SPMS patients (66). Other functions may also be altered in patients with disability progression but may be rather difficult to be precise or to objectify, such as cognitive impairment or psychiatric symptoms (21). Cognitive impairment is suggested to be more frequent in SPMS than in RRMS patients (67, 68).

Different clinical outcome measures have been widely discussed elsewhere as resources for the evaluation of people with MS (4). It is still noteworthy that a regular and standardized documentation of the patients' disease status is needed to enable the use of clinical measures in the follow-up of MS patients.

An EDSS absolute increase of 0.5 or 1.0 point depending on the baseline score is the most commonly used marker for the diagnosis of disease progression. However, the well-known limitations on the responsiveness of this tool and the characteristics of the scale represent an obstacle for its application. In particular, between the EDSS steps of 4.5 and 7.0, this scale is rather a measure of ambulation as this function determines the final score. At upper levels, general quality of life and self-care mark the EDSS steps. So, other disability functions (e.g., cognition, arm function, bowel, and bladder function) may become difficult to assess in patients with EDSS >4.0, which are generally on the SPMS phase of the disease. Patients may present a significant progression of, e.g., visual symptoms, which would not affect the overall EDSS step in patients with limited ambulation. The deterioration of single-function systems, also assessed through the EDSS, should be considered in the future for the detection of clinical progression. This is although still not standard of care in neurological practice.

The MSFC, designed to overcome the EDSS' limitations, is able to characterize progression through functional tests. A relative increase of 20% has also been described as significant in certain subtest parts of the MSFC, such as the T25FW or the 9HPT to detect disability (69–73). However, the lack of a gold standard as an anchor measure makes analysis difficult. This mark was, e.g., significant when the Guy's Neurological Disability Scale (74) was used as a reference, but other studies using the Multiple Sclerosis Impact Scale (75) could not confirm these results (72, 76, 77). In low-contrast visual tests, such as the LCLA, a change of seven-letter reductions could be clinically meaningful and correlated with functional scores and ocular coherence tomography results (78, 79). The Symbol Digit Modalities Test (SDMT), a widely used cognition test, can also be used as an alternative to assess this domain in MS patients (80). A four-point (or 20%) worsening of this latter test could also detect changes beyond the EDSS (81, 82). Nevertheless, these tests may have a high variability and be affected by non-MS-related factors. A regular evaluation with these functional tests is required to recognize, with a high level of reliability at long term, a disease progression.

As gait and balance impairment play a central role in the quality of life of MS patients, it is expected and evidenced that these domains are the most affected (or as so perceived by the patients) in the progressive forms of the disease (34, 83, 84). Techniques involving gait analysis and balance evaluation similarly support the objective evaluation of the patients and early identification of impairment (65, 85, 86).

Nevertheless, though the use of these clinical outcome measures represents a significant support, there is a well-known tool that should never be underestimated: the clinical history. A systematic and oriented anamnesis could be a key anchor in the identification of progressive disease and disease activity. The objectification of chronic or long-persistent changes in the disease status and the ability to discern if these changes have a direct relationship with the underlying inflammatory disease is crucial in SPMS. Follow-up evaluations should include the multidimensionality of the possibilities of disability progression. The disability is the center of the definition of progression and the interrogatory between the physician and patient is probably the most important resource, especially when it is performed on standard basis.

Probably, the collection of individual results for every functional domain for an independent evaluation over time would be a better option than a summary final score for the assessment of progressive MS, although a single score may be more comfortable for the physicians. Through more precise and efficient parameters, the diagnosis and treatment of MS patients has improved and standardized with a great practical benefit. Digital resources and tools are emerging in the field as well.

Automatized software tools may be the key for the continuous interpretation of these results at long-term basis. Certain tools could support a standardization of the anamnesis to direct the identification of progression and objectivize the disease, such as the MS Prediction Score (87), MS Progression Discussion Tool (66), or the SPMS nomogram (88), which can systematically evaluate subtle signs of progressive disease and their influence on the daily tasks. In example, the MS Progression Discussion Tool included the most common demographic and clinical characteristics of RRMS, transitioning, and SPMS patients for the development of a standardized anamnestic tool (66). Not only neurological functioning in each subdomain is assessed by the evaluating physician, but the impact on daily life activities as well. The inclusion of these tools in further trials concerning SPMS patients may illustrate their applicability in daily practice (89, 90).

## Conclusions

Especially through the appearance of treatment options, there is an emerging need of identifying biomarkers that suggest (or predict) a progression of disability in people with MS. Although imaging resources have an increasing importance in the MS management, the clinical evaluation of the patients has a central role in the identification of secondary disability progression. Besides the EDSS, other clinical resources are available on the neurological practice to document a progressive disease. However, a key factor is the regularity and applicability of the tools. The consistent use of user-friendly outcome measures taking advantage of technological resources emerges as a promising possibility for MS specialists. It is probable that digital tools enabling a constant monitoring of neurological function in the daily life could be the clue to identify progression early in the real world (91).

## Author Contributions

HI and TZ participated in the conceptualization of the manuscript. HI and TZ wrote the first draft of the manuscript. UP and KA performed a critical and intellectual revision of the content. KA and TZ participated in the acquisition of funding for the publication. HI designed and edited the figures. All authors contributed to the final revision of the manuscript.

## Conflict of Interest

HI received speaker honoraria from Roche. UP received speaker honoraria from Merck, Biogen and Bayer. UP received additionally personal compensation from Biogen and Roche for consulting service. TZ received personal compensation from Biogen Idec, Bayer, Novartis, Sanofi, Teva, and Synthon for consulting services. TZ received additional financial support for research activities from Bayer, Biogen Idec, Novartis, Teva, and Sanofi Aventis. KA received personal compensation from Roche, Novartis, Sanofi, and Celegene for consulting services.
